# Hepatitis B Virus DNA Integration and Clonal Expansion of Hepatocytes in the Chronically Infected Liver

**DOI:** 10.3390/v13020210

**Published:** 2021-01-30

**Authors:** William S. Mason, Allison R. Jilbert, Samuel Litwin

**Affiliations:** 1 Fox Chase Cancer Center, Philadelphia, PA 19111, USA; Samuel.litwin@fccc.edu; 2 Department of Molecular and Biomedical Science, School of Biological Sciences, University of Adelaide, Adelaide, SA 5005, Australia; allison.jilbert@adelaide.edu.au

**Keywords:** hepatitis B virus, virus DNA integration, clonal expansion, hepatocellular carcinoma, immune-mediated killing, hepatocyte proliferation

## Abstract

Human hepatitis B virus (HBV) can cause chronic, lifelong infection of the liver that may lead to persistent or episodic immune-mediated inflammation against virus-infected hepatocytes. This immune response results in elevated rates of killing of virus-infected hepatocytes, which may extend over many years or decades, lead to fibrosis and cirrhosis, and play a role in the high incidence of hepatocellular carcinoma (HCC) in HBV carriers. Immune-mediated inflammation appears to cause oxidative DNA damage to hepatocytes, which may also play a major role in hepatocarcinogenesis. An additional DNA damaging feature of chronic infections is random integration of HBV DNA into the chromosomal DNA of hepatocytes. While HBV DNA integration does not have a role in virus replication it may alter gene expression of the host cell. Indeed, most HCCs that arise in HBV carriers contain integrated HBV DNA and, in many, the integrant appears to have played a role in hepatocarcinogenesis. Clonal expansion of hepatocytes, which is a natural feature of liver biology, occurs because the hepatocyte population is self-renewing and therefore loses complexity due to random hepatocyte death and replacement by proliferation of surviving hepatocytes. This process may also represent a risk factor for the development of HCC. Interestingly, during chronic HBV infection, hepatocyte clones detected using integrated HBV DNA as lineage-specific markers, emerge that are larger than those expected to occur by random death and proliferation of hepatocytes. The emergence of these larger hepatocyte clones may reflect a survival advantage that could be explained by an ability to avoid the host immune response. While most of these larger hepatocyte clones are probably not preneoplastic, some may have already acquired preneoplastic changes. Thus, chronic inflammation in the HBV-infected liver may be responsible, at least in part, for both initiation of HCC via oxidative DNA damage and promotion of HCC via stimulation of hepatocyte proliferation through immune-mediated killing and compensatory division.

## 1. Introduction: Integration of HBV DNA into the Chromosomal DNA of Hepatocytes

**Figure 1:**
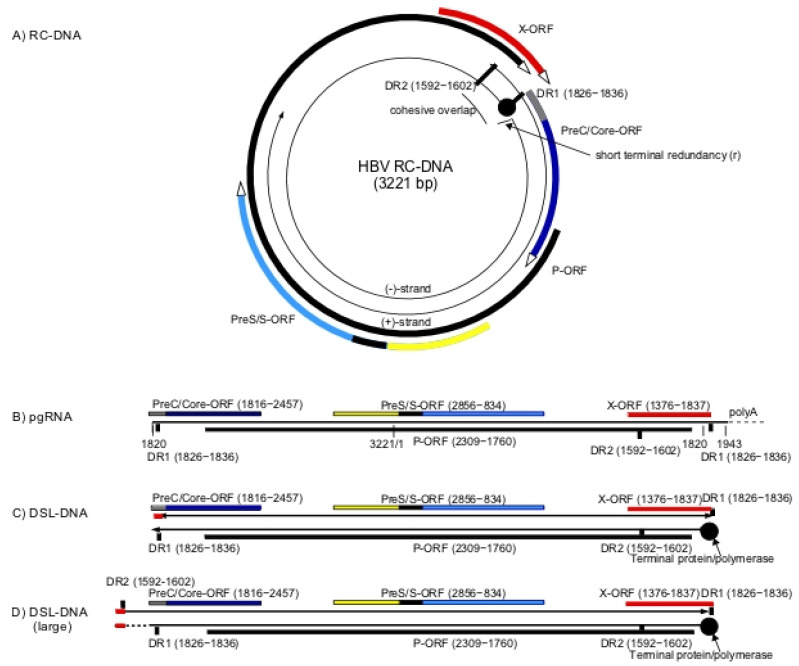
Human hepatitis B virus (HBV) DNA replication intermediates and integration into the chromosomal DNA of hepatocytes: Viral RC-DNA, shown at the top (**A**), is converted to covalently closed circular DNA (CCC-DNA) (not shown) during initiation of infection. All of the virus open reading frames (ORFs) are encoded on the negative strand of the RC-DNA genome as shown and include the polymerase (P-ORF), PreC/Core (C-ORF), PreS/S-ORF and X-ORF. Pre-genomic RNA (pgRNA) (**B**) is transcribed from CCC-DNA and functions as the mRNA for core (nucleocapsid) and polymerase (reverse transcriptase) proteins, both of which are required for viral DNA synthesis. Core protein is also recognized in infected cells as hepatitis B core antigen (HBcAg). An mRNA with extra 5′ sequences is the template for the PreC/Core protein that is processed into the secreted hepatitis B e antigen (HBeAg), while sub-genomic mRNAs encode the HBV envelope proteins (HBsAg) and the HBV X protein (HBx). Pre-genomic RNA along with the viral polymerase is packaged in the cytoplasm into viral nucleocapsids comprised of core protein and is reverse transcribed into new viral DNA. RC-DNA (**A**) is the major product of reverse transcription. As discussed in the text, DSL-DNA (**C**) is a minor species formed when the RNA primer for second strand synthesis fails to translocate from DR1 to DR2. DSL-DNA is the usual precursor for HBV DNA integration into chromosomal DNA. A second larger form of DSL-DNA (**D**) is proposed to be formed from RC-DNA during initiation of infection, via strand displacement DNA synthesis through the cohesive overlap region of RC-DNA. As discussed, this larger DSL-DNA species may also be a precursor for integration of HBV DNA into chromosomal DNA.

Hepadnaviruses have a relaxed circular partially doubled stranded DNA genome (RC-DNA) (Figure 1A) that is converted into a covalently closed circular DNA (CCC-DNA) during initiation of infection [1,2]. CCC-DNA is the template for viral RNA synthesis, including formation of the pre-genomic RNA (pgRNA) and mRNAs for the viral proteins. pgRNA is greater than unit length (Figure 1B) and is reverse transcribed to form virion RC-DNA. In addition, a small fraction of virions contains double-stranded linear DNA (DSL-DNA) (Figure 1C), formed by the reverse transcription pathway when the RNA primer for second strand (DNA dependent) DNA synthesis fails to translocate from DR1 to DR2 to prime formation of RC-DNA [3]. During initiation of infection, this DSL-DNA may be converted to aberrant forms of CCC-DNA by illegitimate recombination [4].

The existence of a another and larger form of DSL-DNA has been inferred from studies of aberrant forms of CCC-DNA [5]. Unlike the shorter form of DSL-DNA, this second DSL-DNA has not been found in virions, and is possibly formed from RC-DNA during initiation of new rounds of infection, via strand displacement DNA synthesis through the cohesive overlap region of RC-DNA (Figure 1A). As a result, this DSL-DNA would have a large terminal redundancy encompassing the cohesive overlap region of RC-DNA (Figure 1D).

These two DSL-DNA forms appear to be the primary substrates for random integration of HBV DNA into the chromosomal DNA of hepatocytes that occurs during the course of infection [4,6,7,8]. Double-strand breaks in chromosomal DNA appear to be preferred sites of integration via illegitimate recombination, with some sequences lost from the ends of the DSL-DNA forms [9]. Among those integrations likely to be derived from DSL-DNAs, approximately 75% appear to be derived from the shorter form (Figure 1C), as integrated HBV DNA sequences frequently have virus-cell DNA junctions near to the left hand and right hand ends of the shorter form of DSL-DNA (Figure 1C) [10,11]. Approximately 25% of integrated HBV DNA sequences have left hand virus-cell DNA junctions in the cohesive overlap region and appear to arise from the DSL-DNA with the large terminal redundancy [11] (Figure 1D). In summary, various lines of evidence suggest that DSL-DNAs are major substrates for random integration of HBV DNA into host chromosomes.

## 2. HBV DNA Integration as a Risk Factor for HCC

The most studied hepadnaviruses fall into two distinct groups, ortho-hepadnaviruses infecting mammals and avihepadnaviruses infecting birds [12]. For the ortho-hepadnaviruses there is evidence that hepadnavirus DNA integration into host DNA is one of the risk factors for development of HCC in HBV-infected humans and woodchuck hepatitis virus (WHV)-infected woodchucks. However, whether there are differences in the relative amounts of RC-DNA and the two forms of DSL-DNA between different HBV strains, and between different hepadnavirus species that impact the frequency of integration and risk of the development of HCC, is not known.

For instance, while viral DNA integration occurs in duck hepatitis B virus (DHBV)-infected ducks (e.g., reference [6]), it is not known to be an HCC risk factor in this species. Instead, in domestic ducks environmental carcinogens (e.g., aflatoxin) appear to be a major risk factor for HCC [13,14]. The absence of elevated levels of HCC in DHBV-infected domestic ducks may also reflect the high prevalence of secondary amyloidosis of the liver, a competing and rapidly developing condition that is common in domestic ducks, that leads to accumulation of extracellular amyloid protein in the liver [15]. Secondary amyloidosis does not correlate with DHBV infection, occurring in both infected and uninfected ducks [16].

In contrast, major involvement of viral DNA integration in HCC emergence is clearly seen in woodchucks with chronic WHV infection, where HCCs typically display transcriptional activation of N-myc2 or, occasionally, N-myc1 or C-myc, due to WHV DNA integration [17]. Transcriptional activation of N-myc2 has been associated not only with nearby integration [17], but also integration at the distal win [18] and b3n loci [19,20]. At least in the case of nearby integration, a WHV enhancer element appears to be responsible for activation of myc transcription [21].

Experiments on the possible role of myc in woodchuck HCCs were presumably stimulated by reports that bursal lymphomas in chickens are due to transcriptional activation of C-myc via promoter insertion of avian leukosis virus (ALV) DNA (provirus) [22,23]. This idea was supported by studies showing that an acute-transforming avian retrovirus, MC29 (myelocytomatosis virus 29) [24], expressed a variant form of the host myc gene [25], reiterating the transforming potential of myc. A model for HCC related to the avian bursal lymphoma model was first suggested by studies showing elevated myc expression in HCCs from WHV-infected woodchucks, sometimes associated with altered transcript sizes [26]. It should be noted, however, that woodchuck HCCs only emerge two to three years after WHV infection of newborns, while bursal lymphomas in ALV-infected chickens become prevalent around the time of sexual maturity. Hence, the sequence of events leading to HCC after transcriptional activation N-myc2 via WHV DNA insertion may be more complicated. The additional steps, if any, needed to fully transform the woodchuck hepatocyte are unknown.

It was initially suspected that oncogene activation via viral DNA integration might also characterize HCCs arising in humans and ground squirrels with chronic hepadnavirus infection. So far, proof of this, as a general rule, remains elusive. While the association of WHV integration with myc activation appears in nearly all woodchuck HCCs, and C-myc expression is elevated in many HCCs arising in ground squirrel hepatitis virus (GSHV)-infected Beechey ground squirrels, the latter is associated with C-myc gene amplification rather than viral DNA integration [27,28]. This difference exists despite the close genetic relationships between both the viruses, WHV and GSHV, and their respective hosts [27,29]. The importance of integrations near myc in woodchuck hepatocarcinogenesis is consistent with reports suggesting that HCC develops more rapidly in woodchucks chronically-infected with WHV [30] than in Beechey ground squirrels chronically-infected with GSHV [31]. This difference may be, in part, virus related, since HCC also develops more slowly in woodchucks chronically infected with GSHV than with WHV [27,29]. This delay also correlates with a lower frequency of GSHV integration in N-myc alleles of the woodchuck HCCs [27]. Thus, while informative, WHV induced HCC via insertional activation of myc may be just a very unique example of much more diverse mechanisms of hepatocarcinogenesis.

HCC in humans chronically infected with HBV also appears to follow more indirect and diverse pathways than seen in the WHV-infected woodchuck. The difficulty in tracking these pathways relates in large part to the long time-lapse between infection and HCC: the incidence of HCC only increases substantially after 40 years of age in those infected with HBV at birth or early in life. Moreover, prior to the introduction of genome wide sequencing protocols, it did not seem that HBV DNA integration had a role in most HCCs, as only a few tumors were found with HBV integration in candidate oncogenes [32,33,34] (erbA and cyclinA). More recently, HCC integrations have been found in TERT, MLL4, CCNE1, and CCNA2, etc. (e.g., [35,36]), mostly in promoter/intron regions, as well as in DNA sequences for non-coding RNAs [37]. However, unlike the situation in the woodchuck model, well-defined relationships between integration and tumorigenesis have been found in less than half of human HCCs analyzed so far. (Genetic causes of human HCC are further addressed in the article by Dr. Zucmann-Rossi).

Like HBV DNA integration, chronic hepatitis is also thought to be a major HCC risk factor in mammals [38]. Chronic HBV infections typically occur at birth from infected mothers, or during the first year of life, and often lead to chronic hepatitis. Inflammation is believed both to promote and to contribute to initiation of hepatocarcinogenesis; this would occur by inducing oxidative DNA damage (initiation) [39] and via stimulation of hepatocyte proliferation through immune-mediated killing and compensatory division (promotion). Thus, even without viral DNA integration, chronic HBV infection might eventually lead to HCC, with integration being an additional risk for hepatocarcinogenesis.

In summary, HBV integration appears to be an HCC risk factor in a process that can employ a variety of pathways, not always including integration-specific activation of a host oncogene. It is also likely that HBV DNA integration, per se, is not a factor in some human HCCs, rather being carried passively in cells that go on to form tumors. That is, chronic inflammation due to HBV infection may be at least as important as HBV DNA integration, if not more so, in hepatocarcinogenesis. Despite the lack of universal associations between human HCC and one or even a few defined integration sites, attempts to determine if HBV DNA integration is relevant to the emergence of all human HCCs persists because essentially all HCCs are clonal with respect to one or more HBV integration sites. While this association reflects the idea that tumors are derived from infected hepatocytes, it still leaves open the question of the absoluteness of a causal relationship between integration and the development of HCC.

Whatever its role, HBV integration per se is not usually the sole cause of an HCC, inasmuch as integration occurs both early and continuously throughout a chronic HBV infection [40,41,42], and presumably during transient HBV infections as well (cf., references [8,40]), while HCC generally emerges only after several decades of chronic infection. While it might be argued the delay is due to a failure of integrations to target particular host genes, it is important to keep in mind that even one integration per 10,000 hepatocytes, as seen in transient WHV infections for example [8], would add up to over 10^7^ integrations in the human liver as a whole. This slow progression, indicating that integration is not usually acutely transforming, has led to the hypothesis that HBV proteins expressed from CCC-DNA and/or integrated DNAs may be pro-carcinogenic, even if, by themselves they are unable to cause acute cellular transformation. Thus, pathways for human HCC in the context of chronic HBV infection may be extremely complicated in that chronic inflammation, integration, normal virus proteins and, perhaps, mutated virus proteins might all contribute to HCC, with different tumors reflecting varying pathways to neoplasia.

## 3. Viral Proteins and HCC

HBeAg and HBcAg are usually not expressed from integrated DNAs, since integration of the shorter form of DSL-DNA separates these genes from their promoter (see Figure 1C) and even integrations within the cohesive overlap at the upstream end of the longer form of DSL-DNA (Figure 1D) would partially ablate regulatory sequences. Perhaps for this reason, HBeAg and HBcAg have not been widely considered to be pro-carcinogenic. In contrast, HBsAg and HBx have downstream promoters that remain intact in DSL-DNA. When CCC-DNA levels decline in late stages of infection, expression of envelope proteins from integrated HBV DNA can become a major source of circulating HBsAg [43]. A C-terminally truncated HBx protein can also be expressed from integrated forms of DSL-DNA (Figure 1C,D). Moreover, HBx-host fusion transcripts (e.g., [44]) have been reported that might have a role in carcinogenesis.

Thus, it has been proposed that mutated variants of both envelope proteins (HBsAg) and C-terminally truncated HBx expressed from integrated HBV DNA, as well as intact HBx made from CCC-DNA, may be oncogenic [45,46,47], albeit slow acting. A problem in testing these proposals is that, as noted, there is no evidence from human studies of rapidly acting viral oncogenes leading to rapid development of HCC, and no easy way of evaluating pathways to human HCCs that may actually evolve over many decades.

Perhaps the most interesting possibility of a role for a viral protein in oncogenesis comes from studies of HBx, a protein required for transcription of CCC-DNA. A main role of HBx in HBV replication is to prevent the host smc5/6 complex from shutting down transcription of CCC-DNA [48,49]. This shutdown is prevented though HBx mediated degradation of one or more of the smc5/6 complex components [48]. Since smc5/6 is involved in host DNA repair and chromosome maintenance, degradation by HBx could over time be carcinogenic. Interestingly, HCC has been observed in selected HBx transgenic mice (e.g., [46]). However, studies of other HBx transgenic lineages found that HBx was not carcinogenic, but could potentiate the effect of a known liver carcinogen (DEN, di-ethyl-nitrosamine) [50,51]. This may support the notion that HBx, via degradation of smc5/6, potentiates carcinogenesis by inhibiting repair of DNA damage caused by DEN, in the transgenic mice and endogenous factors, such as chronic inflammation in the context of chronic HBV infection.

A role for HBsAg proteins in HCC formation, particularly HBsAg proteins with mutations in the PreS2 domain, has also been proposed based on studies in transgenic mice [52,53] and analyses of Type II foci of ground glass hepatocytes (GGH) that can appear in patients with chronic HBV infection [54], as well as patient studies suggesting that these mutations are an HCC risk factor (e.g., references [55,56]).

Type II GGHs have been suggested to be preneoplastic lesions [54]. The histologic appearance of GGH results from over-accumulation of viral envelope protein in the endoplasmic reticulum (ER) [57], which seems from other studies to be toxic to hepatocytes (e.g., [58]). Thus, oncogenic effects of mutated HBsAg in the context of type II GGH may be indirect and result from enhancement of hepatocyte death and compensatory proliferation, rather than ER stress. This remains to be resolved.

In summary, the vast majority of human HCCs are clonal with respect to HBV DNA integration sites, implying that transformed cells emerge from cells into which HBV DNA had already integrated. As far as is known, hepatocytes are the only liver cells that are infected by HBV. Thus, whatever the mechanisms for cell transformation leading to particular HCCs, it is likely that an infected hepatocyte is the ultimate HCC precursor.

## 4. Hepatocyte Proliferation and Liver Maintenance

While chromosomal DNA damage resulting from inflammation and HBV DNA integration, plus hepatocyte death and proliferation induced by immune-mediated killing of infected cells, seem causative in emergence of HCCs, these links to HCC are mostly speculative. This is also the case for attempts to understand the cellular origin of HCCs.

An important but unresolved issue is whether all hepatocytes can potentially give rise to HCCs, or if HCCs arise instead from a subset of hepatocytes with distinct proliferative activity; that is, if promotion following initiation event(s) [59] is built into a subset of hepatocytes in the normal liver.

In the healthy adult liver, hepatocyte turnover (hepatocyte death and replacement by proliferation of surviving hepatocytes) is often assumed to be very low, about 0.01%–0.1% per day [60]. These numbers are consistent with our observations of PCNA (proliferating cell nuclear antigen) labeling indices in tissue sections of uninfected woodchuck liver [61], assuming that S-phase is about 8 h and that PCNA staining therefore reflects about one-third of the cells in S-phase over a 24 h period. (PCNA nuclear staining is characteristic of cells in S-phase). However, PCNA staining indices of 0.4% and more in “normal” human liver have also been reported, suggesting the level of hepatocyte turnover in infected human liver may be higher than thought [62,63,64]. The issue of hepatocyte proliferation rates may be critical in attempts to evaluate models of hepatocyte homeostasis and changes in complexity of the hepatocyte population over time, particularly whether all hepatocytes contribute to liver homeostasis or if this role is restricted to a subset of hepatocytes.

**Figure 2:**
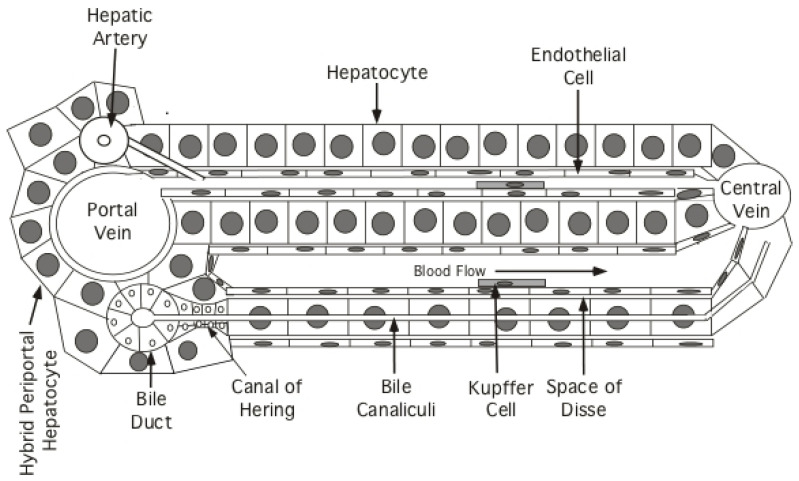
Structure of the hepatic lobule: schematic, essentially one dimensional view, of the hepatic lobule, illustrating a few tracts of hepatocytes and supporting cells extending from a portal vein (in the portal triad) to a central vein. Hepatocytes or a subset of the hepatocytes constituting the limiting membrane around the portal triads (hybrid periportal hepatocytes), have the proliferative capacity to respond to severe liver injury and restore the hepatocyte population of the lobule [66]. Arterial and venous blood enters the sinusoids of the lobule at a portal triad and is collected at a central vein. Bile is secreted from hepatocytes into bile canaliculi, formed at the junction of adjacent hepatocytes, and flows into the bile ducts. The sinusoids are lined with a fenestrated endothelium; whether the fenestrations are large enough to allow free passage of HBV from the blood to hepatocytes is controversial, with an alternate suggestion that virus is actively transported from the blood stream to hepatocytes [71]. Kupffer cells (liver macrophages) are also shown, as well as the cells that form the Canals of Hering, which facilitate transport of bile from canaliculi to the bile ducts and have also been proposed to be hepatocyte progenitor cells [67]. Hepatic stellate cells (not shown), found within the Space of Disse, are multifunctional and, in the context of chronic HBV infection, are responsible for the development of cirrhosis in response to chronic liver injury. Figure adapted from Jilbert et al., 2008. Pathogenesis of HBV Infections, Chapter 7: HBV Human Virus Guide, 2nd Ed, CL Lai and S Locarnini Eds. Reproduced with permission from International Medical Press.

Based mostly on experiments in mice, there are at least four models for how new hepatocytes can arise in the hepatic lobules (Figure 2). Model (1): new hepatocytes form from progenitors at the portal triad and flow towards the central vein [65,66]; Model (2): new hepatocytes arise at the central vein and flow towards the portal triad [67]; Model (3): a subset of telomerase-positive hepatocytes (~3%) scattered throughout liver lobules differentially proliferate to maintain liver mass [68]; Model (4): all hepatocytes can proliferate to maintain liver mass [60,69,70].

Historically, studies of hepatocyte proliferation using, for example, nuclear PCNA as a histologic marker of S-phase hepatocytes, have found proliferating hepatocytes scattered at random throughout the hepatic lobule, without an obvious zonation. It has also been argued from recent experiments that both polyploid and diploid hepatocytes proliferate to contribute to liver renewal [60,69,70].

These and related studies appeared to rule out the need for an essential hepatocyte progenitor at the portal triad [65] except in cases of severe liver injury mediated by toxic chemicals (e.g., carbon tetrachloride) [66] (Model 1). In these latter studies, “hybrid periportal hepatocytes” were discovered that have a survival advantage, possibly because they fail to express high levels of de-toxifying enzymes [66]. Under cases of extreme injury, they can regenerate a major fraction of the hepatic lobule [66]. These hepatocytes are found at the limiting plate surrounding the portal triad and, unlike other hepatocytes, express low levels of Sox9. While these hybrid periportal hepatocytes can proliferate and stream from the portal triad to replace damaged hepatocytes during severe injury, the idea that all hepatocytes originate at the portal tract and stream towards the central vein during normal liver homeostasis or in the context of chronic HBV infection does not appear likely, as shown in reference [66] for normal liver homeostasis. First, severe acute injury is rare and, second, hepatocytes scattered throughout the lobule appear to proliferate during chronic HBV infection. Thus, expansion of the hybrid periportal hepatocytes may have resulted because, with a survival advantage, they were the only hepatocytes able to proliferate to restore liver homeostasis after massive hepatocyte damage [66].

A more problematic issue in liver homeostasis is raised by the report that all hepatocytes arise from the layer of hepatocytes surrounding the central vein [67] (Model 2). In this study, the authors took advantage of the fact that axin2 is predominantly expressed in pericentral hepatocytes. Thus, a Tamoxifen regulated cre recombinase expressed under control of an axin2 regulatory sequence in transgenic mice should differentially activate the fluorescent transgene in the pericentral hepatocytes of ros26-floxed mice following injection of Tamoxifen. (Tamoxifen facilitates nuclear import of the cre recombinase, which was engineered to contain an estrogen receptor; this recombinase is restricted to the cytoplasm in the absence of Tamoxifen). Wang et al. [67] argue that hepatocytes at the central vein, in which ros26 is activated by the recombinase following Tamoxifen administration, might eventually expand to replace all hepatocytes in the lobule, though an average of only 40% replacement was observed after one year [67].

The conclusion that central-vein hepatocytes are responsible for hepatocyte homeostasis has been challenged in more recent studies, which do not support all of the earlier conclusions [72,73]. In view of these issues, additional support for the original claim in reference [67] would be helpful. At this point, the idea that all or most new hepatocytes originate at and flow from the central vein does not appear compatible with the results of other studies, which suggest that hepatocyte replacement within each of the three traditional lobular zones (periportal, mid, and pericentral) is adequate to maintain normal liver mass [60].

Alternatively, it has been reported that hepatocyte renewal is dependent upon a subset of telomerase-positive hepatocytes scattered throughout the liver lobule [68] (Model 3). In particular, mice transgenic for cre recombinase expressed under control of the TERT locus were exposed to a Tamoxifen pulse. This led to fluorescent marker activation in about 3% of hepatocytes. Cell sorting established that these hepatocytes had elevated TERT mRNA (TERT-high) as compared to unlabeled hepatocytes. In addition, 5-ethynyl-2′-deoxyuridine (EdU) labeling of S-phase hepatocytes indicated that TERT-high hepatocytes had about a six- to seven-fold replication advantage over hepatocytes with a low-TERT mRNA (TERT-low) phenotype. Finally, the fluorescently-labeled hepatocyte population expanded about 10-fold in the course of a one-year follow-up, giving rise to both TERT-high and TERT-low hepatocytes; that is, to maintain the TERT-high population at 3%, one of the progeny cells of a TERT-high hepatocyte is usually a TERT-low hepatocyte. TERT-high hepatocytes would only give rise, presumably, to two TERT-high progenies if needed to maintain the TERT-high population.

Thus, by Model 3 the hepatocyte population is maintained primarily through division of a small population of TERT-high hepatocytes that stays approximately constant in size (3%) in full grown mice. What remains unclear is the role of this population in liver growth in immature mice and the flexibility of this TERT-high population for self-renewal when TERT-high cells die off due, for instance, to immune-mediated killing as in the case of chronic HBV infection. That is, are there innate mechanisms for maintaining a fixed fraction of hepatocytes in this TERT-high state? In addition, the proliferative potential of the pulse-labeled, TERT-high hepatocytes should probably be compared to TERT-low hepatocytes in hepatocyte transplantation experiments, to see if the TERT-high population can be regenerated from TERT-low hepatocytes. In brief, this model is provocative but, so far, incomplete.

Finally, Model 4 in which most or all hepatocytes have the capacity to contribute to homeostasis appears most consistent with published data. However, it is currently difficult to rule out Model 3 since, in many respects, it may give results similar to those from Model 4. This is discussed in greater detail below.

Irrespective of how this issue is resolved, it seems apparent that persistent immune-mediated killing and compensatory proliferation in the hepatocyte population will lead to its genetic narrowing together with its increased clonality. Clonal expansion of hepatocytes is another presumed HCC risk factor, as it may facilitate the emergence of large hepatocyte clones with preneoplastic changes, including the ability to evade the antiviral immune response and/or to proliferate more rapidly in response to growth signals.

## 5. Clonal Expansion of Hepatocytes during Chronic HBV Infection

Chronic HBV infections are considered to progress through at least four stages: (1) the immune tolerant (IT) HBeAg-positive stage, with normal liver function enzymes, e.g., alanine amino transferase (ALT), and high virus titers, typically of the order of 10^9^ HBV DNA genomes per ml of serum, suggesting low immune reactivity to infected hepatocytes; (2) the immune active, HBeAg-positive stage, with high or somewhat reduced virus titers and elevated ALT, indicative of active liver disease with immune-mediated killing of infected hepatocytes; (3) the immune active, HBeAg-negative stage, with lower virus titers, but generally >10^4^ HBV DNA genomes per ml, and in some cases elevated ALTs (virus titers >10^4^ per ml are usually taken as evidence of active hepatitis in these patients even if ALTs are normal); (4) the inactive, HBeAg-negative stage with low virus titers, <10^4^ HBV DNA genomes per ml and normal ALTs. Reactivation sometimes occurs in patients in this stage.

As expected from the closed nature of the hepatocyte population, clonal expansion of hepatocytes occurs during the various phases of chronic HBV infection including the IT phase. Using randomly integrated HBV DNA to distinguish and quantify hepatocyte clones, it was found that hepatocyte clone sizes in chronically infected but non-cirrhotic patients range up to tens of thousands of hepatocytes (e.g., references ([10,11,74,75])). Hepatocyte clone sizes can be much larger in cirrhotic nodules, and are even detectable by Southern blot hybridization analysis [76,77] (i.e., containing >100,000 hepatocytes). In the following discussion, however, we focus on the non-cirrhotic liver, since cirrhosis is a characteristic but not universal feature of late stages of chronic HBV infection, while clonal expansion of hepatocytes is characteristic of all stages. It should be noted however that cirrhosis, like the chronic inflammation from which it arises, is also an HCC risk factor. Among those dying of HCC, greater than 50% are thought to be cirrhotic (e.g., reference [78]).

The four models of liver renewal discussed above provide varying scenarios for clonal expansion of hepatocytes by postulating either that all hepatocytes proliferate, or the existence of a subset(s) of hepatocytes that are differentially involved in liver homeostasis. A key feature of all these models is that they do not require a role for a non-hepatocyte precursor to maintain the liver, except perhaps in cases of extreme injury when for instance bile duct cells may trans-differentiate into hepatocytes [79]. Thus, clonal expansion of hepatocytes in chronic HBV infection, where fulminant injury is rare, is presumably due to the proliferation of existing hepatocytes. Hence, with greater disease severity, there will be more genetic narrowing of the liver, loss of complexity of the hepatocyte population and more clonal expansion as some hepatocyte lineages die off and others expand. As noted, this may facilitate emergence of preneoplastic lineages, including those that have not yet acquired a growth advantage over other hepatocytes.

**Figure 3:**
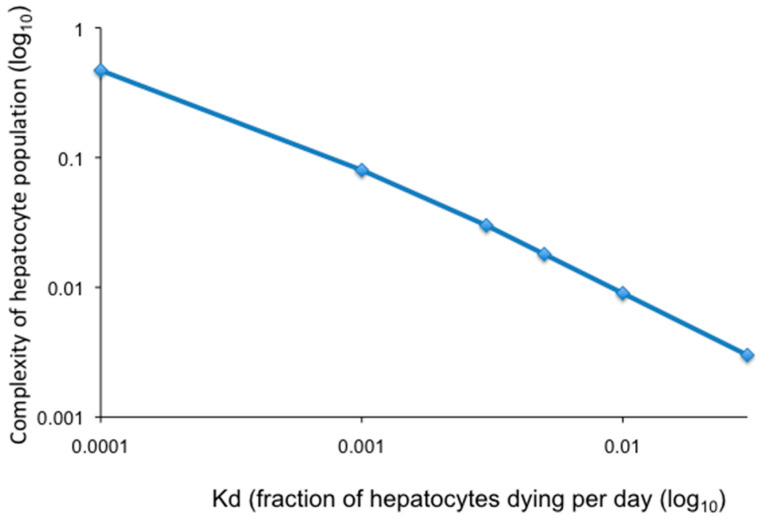
Changes in the complexity of the hepatocyte population after 30 years where all hepatocytes contribute equally to homeostasis (Model 4). Loss of complexity of the hepatocyte population was calculated using the program comp10 [11], assuming the indicated fraction of hepatocytes dying per day (Kd) over a period of 30 years, beginning when the liver reached full size. Declines in complexity are relative to the complexity when the liver first reaches full size. Calculations were made for Kd = 0.0001, 0.001, 0.003, 0.005, 0.01, and 0.03, respectively, as indicated by the diamonds in the plot.

Modeling of loss of hepatocyte complexity (total number of hepatocyte clones/total hepatocytes) as a function of random hepatocyte death and proliferation is illustrated in Figure 3 where all hepatocytes contribute equally to homeostasis as described in Model 4 (above). Calculations were made assuming the indicated fraction of hepatocytes dying per day over a period of 30 years, the approximate time between host maturity and a major increase in the incidence of HCC. Hepatocytes are assumed to be randomly killed and to proliferate at an equal rate to maintain liver size. As shown, a hepatocyte death rate of 0.1% per day (equivalent to a fraction of hepatocytes dying per day (Kd) of 0.001) would reduce the complexity of the hepatocyte population to 0.1 of the complexity at the start (a 10-fold reduction in complexity). Increasing the hepatocyte death rate to 0.5% per day (Kd = 0.005) would reduce the complexity of the hepatocyte population to 0.02 (a 50-fold reduction in complexity). Thus, as the daily rate of hepatocyte death increases, the complexity of the population decreases, leading to genetic narrowing of the liver.

In addition, chronic HBV infection could also be associated with selective emergence of clones of variant hepatocytes, for instance 1) hepatocytes that have a survival advantage because they have either lost the ability to support productive viral infection or are deficient in presentation of HBV antigens and can thereby evade the host immune response, and 2) hepatocytes that have a growth advantage, either because it is an inherent property or due to adaptive cellular mutations [68,80]. Selective processes will increase hepatocyte clone sizes in the population, as is documented for example in rodent models of chronic liver diseases such as hereditary tyrosinemia (e.g., references [81,82] for review).

The emergence of hepatocyte clones with a survival advantage is suggested by the observation that in late stages of chronic HBV infection of humans and chimpanzees only a small fraction of hepatocytes is still productively infected, as assessed by immunohistochemical staining for HBcAg and/or in situ hybridization for cytoplasmic HBV DNA [11,83,84,85], while the majority of hepatocytes no longer appear to produce HBV. Whether the phenotype of these otherwise histologically normal but virus-negative hepatocytes reflects genetic, or epigenetic, changes contributing to evasion of host antiviral immunity is unknown.

**Figure 4:**
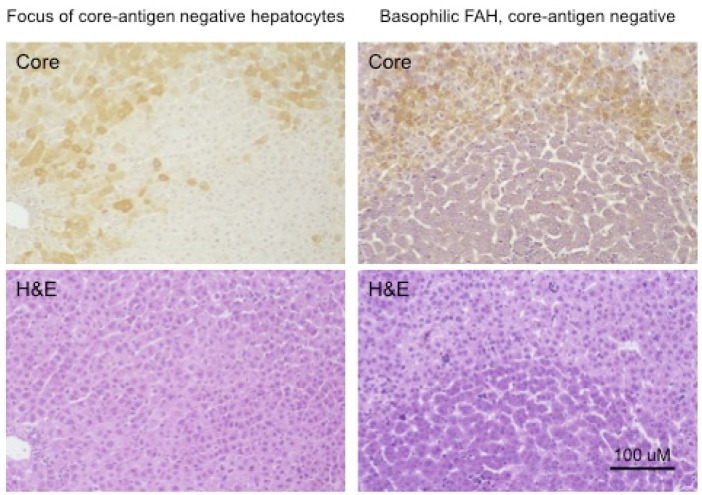
Foci of normal and basophilic, woodchuck hepatitis-virus (WHV) core antigen-negative hepatocytes in chronically WHV-infected woodchuck liver. The left-hand panels are consecutive sections of WHV-infected liver, either immune-peroxidase stained using antibodies to WHV core antigen (top) or Hematoxylin and Eosin (H&E) stained (bottom), showing a focus of histologically normal, WHV core antigen-negative hepatocytes. The right-hand panels are similar consecutive sections showing a basophilic focus of altered hepatocytes (FAH) that are also WHV core antigen-negative. Figure adapted and reproduced with permission from reference [91], Xu et al., Virology, published by Elsevier, 2007.

The change in the hepatocyte population to a less virus-productive state is consistent with the trend to a progressive drop in virus titers during the course of chronic HBV infection. Early in infection, the virus appears to infect the majority of hepatocytes, at least in chimpanzees, where early stages of HBV infection have been most thoroughly studied [86,87,88,89,90]. This is typically followed by a decline in the percentage of infected hepatocytes during the long course of chronic HBV infection. To a small extent this decline in infected hepatocytes is also observed in chronically WHV-infected woodchucks, which have a much shorter life span of about three years. Figure 4 (adapted from reference [91]) shows in the left-hand panels a focus of WHV core antigen-negative, but otherwise normal appearing hepatocytes, late in WHV infection of a woodchuck with high titer viremia. Thus, the cells are apparently resistant to WHV infection and/or expression. In addition, the appearance of these cells in a focus suggests that they represent a single hepatocyte clone. (It should be noted that foci of histologically altered hepatocytes (FAH) which are WHV-negative, and display elevated expression of N-myc [92], are common in the chronically WHV-infected woodchuck [92,93,94], as illustrated in Figure 4, right-hand panels).

**Figure 5:**
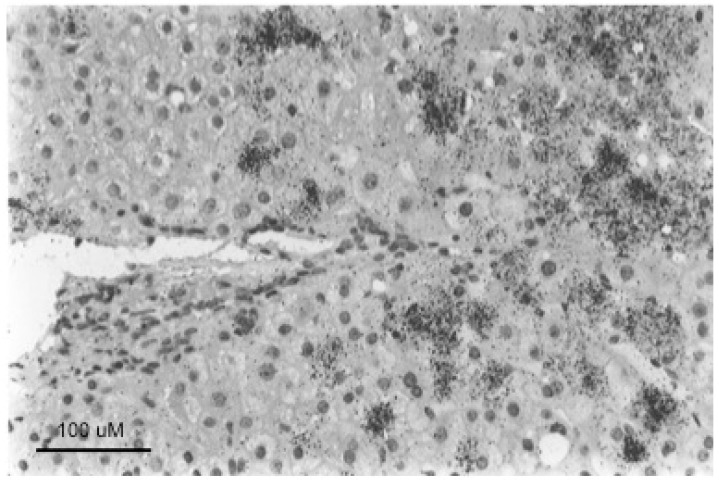
In situ hybridization detection of cytoplasmic HBV DNA, a marker of productive HBV infection in a patient with mild hepatitis and developing cirrhosis. Hepatocytes containing high-levels of HBV DNA were distributed randomly and in periportal regions of the lobule. Many hepatocytes with undetectable levels of HBV DNA were also observed. HBV DNA was detected by in situ hybridization with an ^125^I-labelled HBV DNA probe followed by autoradiography for 168 h and staining with hematoxylin and eosin (H&E). Figure adapted and reproduced with permission from reference [95], Mason et al., Hepatology International, published by Springer, 2007.

Well-defined foci of hepatocytes, negative for HBV replication, as assessed by HBcAg staining of tissue sections, have also been observed in chronically HBV-infected chimpanzees [11]. We are not aware of reports of such foci in human livers, where the number of productively infected hepatocytes in late stages of chronic infection may be too low to show well-defined foci of HBV-negative cells, as illustrated in Figure 5. In any case, while it seems likely that woodchuck FAH are clonal, an obvious question is whether foci of virus-negative but normal appearing hepatocytes as seen in HBV-infected chimpanzees and WHV-infected woodchucks are also clonal, or have another explanation, such as highly localized immune clearance and control. At present, the only point that has been addressed, using sections of HBV-infected human liver, is that hepatocyte clones can be made up of normal appearing and focally accumulated hepatocytes [10,74]. In these examples, clonality was recognized by end-point dilution of liver DNA extracted from histological tissue sections followed by inverse-nested PCR to detect virus-cell junctions. Unfortunately, these samples lacked the well-defined foci of virus-negative hepatocytes seen in woodchucks and chimpanzees that might have allowed a positive correlation between clonal expansion of hepatocytes and the loss of virus production.

A correlation in time between clonal expansion of hepatocytes and loss of virus production might be inferred in any case because, as noted above, the hepatocyte population appears to be self-renewing, so loss of some hepatocyte lineages and expansion of others, including rare hepatocytes not expressing HBV, would be inevitable, especially in the face of an active antiviral immune response. If all hepatocytes can proliferate and hepatocyte death were essentially random, then clone sizes would increase at predictable rates that would depend upon increase in liver size during childhood and the amount of hepatocyte death and compensatory proliferation at all ages.

**Figure 6:**
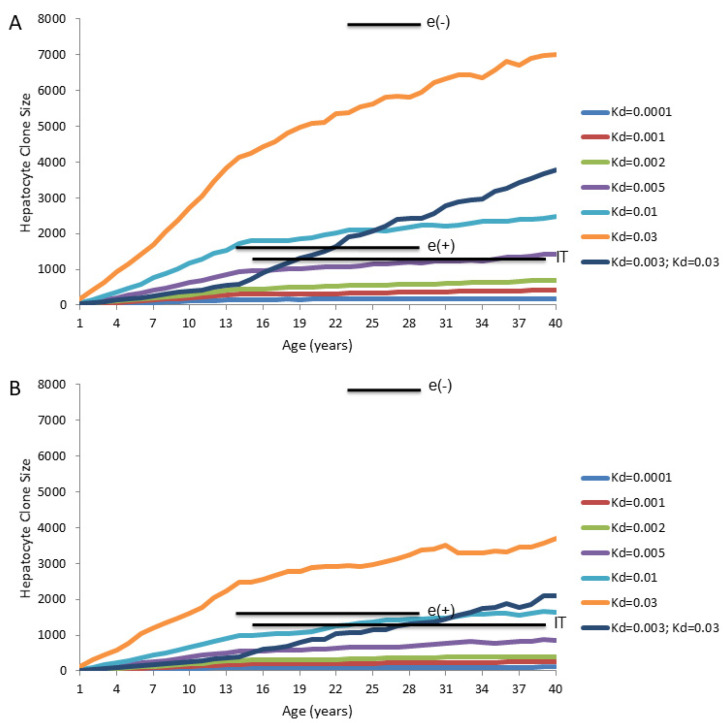
Computer modeling of the predicted emergence of hepatocyte clones in chronically HBV-infected human liver if all hepatocytes are able to proliferate (Model 4): Calculations were made using the program csize8, which was previously described [75]. This program calculates clone size assuming that all hepatocytes are able to proliferate, at random, to maintain liver mass, and that cells are killed at random, with the daily death rates as shown at the right (e.g., Kd = 0.001 means that 0.1% of hepatocytes die per day, and are replaced by compensatory proliferation of surviving hepatocytes). Liver size is assumed to increase 10-fold between birth and maturity at age 14. All hepatocytes (initially 800,000) are assigned unique identifies at birth. In one calculation, the hepatocyte death rate was assumed to be 10-fold lower (0.3% per day; Kd = 0.003) during the first 14 years than after age 14 (3.0% per day; Kd = 0.03). All curves are averages of 10 simulations, with output collected at yearly intervals. The horizontal bars show the geometric mean of maximum hepatocyte clone sizes observed using inverse-nested PCR assays for integrated HBV DNA in a study of three different groups of patients with chronic infection: immune tolerant (IT), immune active, HBeAg-positive (e(+)), and immune active, HBeAg-negative (e(-)) [75]. The bars span the ages covered by each group of patients. (**A**) Predicted maximum clone sizes, assuming 100% of hepatocytes had unique lineage-specific markers at birth. (**B**) Predicted maximum clone sizes if only 1.0% of hepatocytes had detectable lineage-specific markers (e.g., randomly integrated HBV DNA) at birth.

We used a computer model [75] to predict the emergence of hepatocyte clones in the chronically HBV-infected liver. The initial objective was to determine if Model 4 could explain the clone sizes detected using inverse-nested PCR assays for integrated HBV DNA. The predicted maximum hepatocyte clone sizes are shown in Figure 6A. In these calculations, the following assumptions were made: (1) hepatocytes have unique identifiers at birth, (2) the liver increases 10-fold in size between birth and age 14 years, and remains constant in size thereafter, and (3) all hepatocytes die and proliferate at random throughout this process, and none have a selective growth or survival advantage.

Results for predicted maximum hepatocyte clone sizes at different ages are shown for daily hepatocyte death rates of 0.01%, 0.1%, 0.5%, 1% and 3% (equivalent to Kd of 0.0001, 0.001, 0.005, 0.01, 0.03, respectively), which were assumed to be maintained from birth. Mean sizes of the largest hepatocyte clones observed experimentally using assays for integrated HBV DNA in non-cirrhotic liver are also shown for comparison [75].

Interestingly, even for a high and constant hepatocyte death rate (e.g., 0.5% per day, Kd = 0.005) the maximum computed hepatocyte clone sizes are less than the maximum clone sizes experimentally observed in chronically HBV-infected patients using inverse-nested PCR assays for integrated HBV DNA (Figure 6A). This is particularly true in the immune active, HBeAg-negative patients [75]. For these patients, the maximum clone sizes that were observed by inverse-nested PCR assays for integrated HBV DNA exceeded computed sizes even assuming a constant death rate of 3.0% per day (Kd = 0.03) (Figure 6A).

The difference between measured and predicted clone sizes is even more dramatic if patients are assumed to have a lower rate of hepatocyte death during the liver growth phase (0.3% per day; Kd = 0.003) than at maturity at 14 years of age (3.0% per day; Kd = 0.03) (Figure 6A).

It should also be noted that, for IT patients, with normal ALTs and presumed normal liver, the maximum size of hepatocyte clones can exceed that expected from a hepatocyte death rate in the range of 0.01–0.1% (Kd = 0.0001–0.001), often considered to be the background rate of hepatocyte death. This suggests that immune mediated death of hepatocytes is ongoing even in these IT patients [75].

However, as discussed earlier, there is uncertainty about the rate of hepatocyte turnover in the normal human liver, as deduced from PCNA staining indices. Thus, while the size of the larger hepatocyte clones observed in IT patients would not, by our computer modeling, be expected with a death rate of 0.01–0.1% per day (Kd = 0.0001–0.001), the difference from the computer modeling narrows considerably if the actual death rate is as high as 0.5% per day (Kd = 0.005).

It should be noted that Figure 6A gives an idealized prediction of maximum clone sizes, inasmuch as it assumes that all hepatocyte clones are detectable. In fact, in the study in reference [75] which gave rise to the inverse-nested PCR results shown in Figure 6A, the HBV integration frequencies were in the range of 1 copy of integrated HBV DNA per 10^2^–10^3^ cells (0.1–1.0% of hepatocytes contain integrated HBV DNA) at biopsy, as detected by inverse-nested PCR. Thus, at least 99% of hepatocyte clones might not be detected by this approach. The effect of this is to lower predicted maximum clone sizes (Figure 6B) since, on average, many large clones may lack integrated HBV DNA, as detectable by inverse-nested PCR, as a lineage-specific marker. The difference between computer modeled and observed maximum hepatocyte clone size becomes more apparent when we assume that only 1.0% of hepatocyte lineages were detectable (Figure 6B).

While it may be true for all three groups of patients included in Figure 6, it seems most convincing that, for the immune active, HBeAg-negative patients, the larger hepatocyte clone sizes reflect, at least in part, that either a) emergence of hepatocyte clones is selective rather than random (e.g., variants that can avoid immune attack by, for instance, reducing or shutting down virus replication) or b) only a subset of hepatocytes actually participate in liver maintenance during chronic HBV infection (for example, Model 3 in which a small subset of TERT-high hepatocytes has a major growth advantage). As noted earlier, we believe there is more support for the former idea, though this could change if additional evidence supports the notion that a few percent of hepatocytes have a selective growth advantage due to high expression of TERT [68], or for some other reason. In any case, while Model 4 may adequately explain homeostasis of normal liver, it does not adequately explain the detection of the large hepatocyte clones seen during chronic HBV infection.

**Figure 7:**
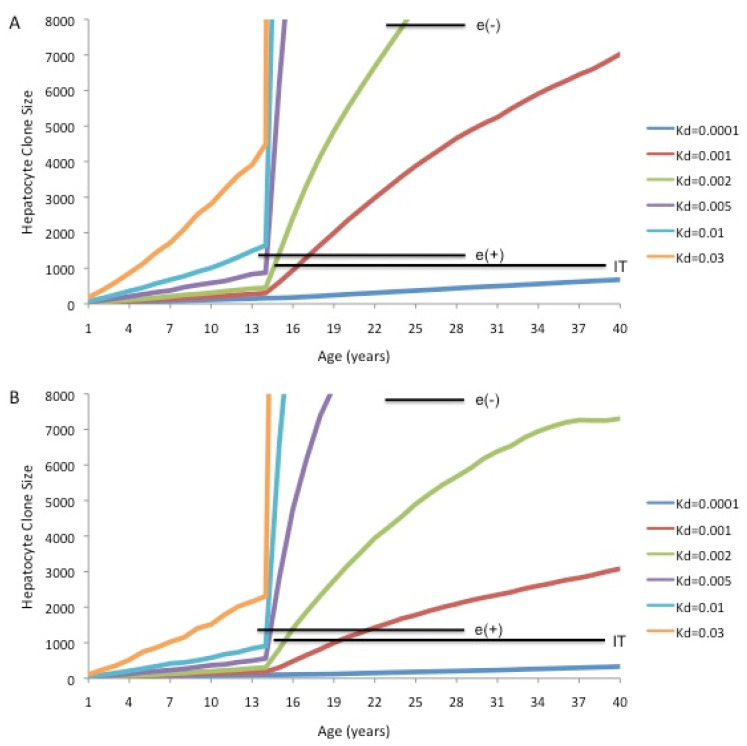
Computer modeling of maximum predicted hepatocyte clone sizes if homeostasis in the adult liver is maintained by TERT-high hepatocytes (Model 3): Maximum hepatocyte clone sizes were calculated for the indicated daily death rates (e.g., 0.2% per day, Kd = 0.002) using the Fortran program ncsize8hp (available upon request to Samuel.litwin@fccc.edu). TERT-high hepatocytes were assumed to represent 3% of the hepatocyte population throughout. During the growth phase of the liver, the TERT-high hepatocytes were assumed to be a self-contained population that did not differentially contribute to liver homeostasis. Once the liver reached full size, they were assumed to have a seven-fold growth advantage over TERT-low hepatocytes, giving rise to both TERT-high and TERT-low hepatocytes [68]. Both TERT-high and TERT-low hepatocytes were assumed to die at the indicated daily rates. The horizontal bars show the geometric mean of maximum hepatocyte clone sizes observed using inverse-nested PCR assays for integrated HBV DNA in a study of three different groups of patients with chronic infection: immune tolerant (IT), immune active HBeAg-positive (e(+)), and immune active HBeAg-negative (e(-)) [75]. The bars span the ages covered by each group of patients. (**A**) Maximum clone size predictions assuming 100% of hepatocytes had lineage-specific markers at birth. (**B**) Maximum clone size predictions assuming only 1.0% of hepatocytes had detectable lineage-specific markers (e.g., randomly integrated HBV DNA) at birth.

To examine the potential effect on maximum hepatocyte clone size if a small fraction of hepatocytes has a dominant role in liver homeostasis, for example as in Model 3, we have performed computer modeling of hepatocyte clone sizes assuming that all hepatocytes contribute to liver growth during maturation, but that homeostasis in the full size liver is predominately due to proliferation of a TERT-high subpopulation of hepatocytes representing a constant 3% of the total hepatocyte population. Figure 7A shows predictions of maximum hepatocyte clone size assuming that TERT-high hepatocytes, constituting about 3% of the hepatocyte population, have a seven-fold growth advantage over TERT-low hepatocytes in adults [68], and that 100% of hepatocytes have detectable lineage-specific markers.

Using Model 4 (Figure 6), we found that maximum hepatocyte clone sizes measured using inverse-nested PCR assays for integrated HBV DNA were generally larger than predicted sizes for a range of hepatocyte death rates, which in our view supports the notion that the larger clones arose from cells with a selective growth or survival advantage. In contrast, Model 3 (Figure 7) shows a much greater sensitivity of predicted clone size to changes in hepatocyte death rates, especially in the range often considered to be indicative of a healthy liver (0.01–0.1% per day, Kd = 0.0001–0.001). At higher death rates, maximum hepatocyte clone size estimates vastly exceed, to our knowledge, hepatocyte clone sizes reported to date in the non-cirrhotic liver. This is true to a significant extent even if only 1% of hepatocytes had detectable lineage markers (Figure 6B and Figure 7B). In reality, however, there may be physical constraints on clonal expansion within a lobule.

In summary, assuming that all hepatocytes contribute to liver homeostasis in normal liver (Model 4) leads to the conclusion that the largest hepatocyte clones that emerge during chronic HBV infection must have a growth and/or survival advantage over most other hepatocytes (Figure 6), suggesting that in the case of chronic HBV infection Model 4 is not strictly true. Experimental observations with liver samples from HBV-infected humans (Figure 5) and chimpanzees and WHV-infected woodchucks (Figure 4) suggest that a shutdown of virus replication may contribute to clonal expansion of hepatocytes by providing a survival advantage over hepatocytes that continue to support the replication of HBV. This would occur even without a proliferation advantage for these hepatocytes.

In contrast, Model 3 provides a scenario for growth of very large hepatocyte clones without the need to evoke a survival advantage, although, as in Model 4, maximum clone size is still correlated with hepatocyte death rates. However, it also seems to suggest that very low levels of hepatocyte turnover would lead to the emergence of very large clones, as TERT-high cells are reported to have a seven-fold growth advantage [69]. Whether this is a realistic model remains to be determined. As noted, the prediction of, in our view, excessively large hepatocyte clones hinges in part on the reliability of hepatocyte turnover (death/proliferation) estimates, which are uncertain. It will be important to know if Model 3 is substantiated in other mouse studies and for the human liver.

Thus, for now we favor Model 4, where all hepatocytes contribute to normal liver homeostasis, as a plausible-starting point for interpretation of both molecular and histological data. However, as noted, this model does not explain the largest clones of hepatocytes that emerge during chronic HBV infection. In this situation, larger hepatocyte clones would need to have growth or survival advantages.

## 6. Summary

Practical Issues: Clonal expansion of hepatocytes is an implicit feature of liver biology which is driven by liver growth during childhood. An additional contributor, both in children and adults, is hepatocyte killing by random or specific injuries. Because the hepatocyte population is self-renewing, some hepatocytes lineages will ultimately be lost while others will expand to maintain liver size. Clonal expansion of hepatocytes will be particularly deleterious for the host if some clones that expand have already acquired genetic or epigenetic changes that predispose to neoplasia. The fact that maximum hepatocyte clone sizes increase as infection progresses through the stages of chronic hepatitis (Figure 6) reinforces one way in which disease stage has a deleterious effect on the liver. An increasing loss in complexity of the hepatocyte population also means that hepatocytes that are preneoplastic have an enhanced likelihood of being in clones that are too large to be readily eliminated by random events. Thus, by this view, chronic HBV infection is both initiating (by HBV DNA integration, oxidative DNA damage, HBx degradation of smc5/6, etc.) and promoting (by immune-mediated killing, with compensatory proliferation) hepatocarcinogenesis. It will probably be beneficial to abrogate this progression as early as possible via antiviral therapy [96], which is known to reduce or eliminate liver inflammation and the short term risk of HCC (e.g., reference [97]).

Theoretical Issues: One major problem in addressing the role of clonal expansion of hepatocytes and its relationship to HCC in HBV patients is a lack of consensus on liver homeostasis in normal liver and during mild liver disease. That is, do all hepatocytes have similar abilities to proliferate to replace those that die, or are they specialized subsets of hepatocytes that do most of the work and, if so, where are they located in the hepatic lobule [66,67,68,98]?

A second major problem is a lack of consensus on hepatocyte turnover rates in normal liver and during the different stages of chronic HBV infection. PCNA staining provides a measure of S-phase hepatocytes and therefore, presumably, of variation in hepatocyte turnover rates with disease progression. However, without knowing the duration of the hepatocyte cell cycle, it is not obvious how to convert PCNA staining indices to daily rates of hepatocyte proliferation and, by inference, daily rates of hepatocyte death. Thus, while we can make predictions on clonal expansion of hepatocytes vs. hepatocyte death/proliferation rates, as in Figure 6 and Figure 7, it is important to keep in mind that actual rates are elusive, and ours are just our own estimates.

The third major problem is the difficulty of correlating clonal expansion of hepatocytes with specific phenotypes that facilitate immune escape. While we favor the idea that loss of productive HBV infection, providing at least partial escape from the antiviral immune response, is a major facilitator of clonal expansion (Figure 4 and Figure 5), this is just an inference made from the various observations discussed earlier. Testing this idea is challenging because of the difficulty of acquiring suitable samples, with well-defined foci of, for instance, HBcAg-negative hepatocytes (cf., Figure 4), and with some lineage-specific marker of clonality such as integrated HBV DNA. While the woodchuck might be a suitable model, it is not clear if the frequency of integration of WHV DNA is high enough to make this practical. Clonal expansion of hepatocytes has been detected in WHV-infected woodchucks by end-point dilution inverse-nested PCR [99], but was only apparent in a few percent of hepatocytes by this assay. Clonal expansion of hepatocytes has been demonstrated using tissue sections of HBV-infected human liver originally cut for histologic analyses [10,74] but, as noted above, we are not aware of reports showing well-defined foci of HBcAg-negative hepatocytes. Thus, a more sophisticated approach may be needed, possibly using other genetic or epigenetic markers of clonality than integrated HBV DNA.

Finally, while numerous, and to us convincing, arguments can be made that HCCs arise from mature hepatocytes (i.e., that contain integrated HBV DNA and were once infected by HBV), it is much less clear if the hepatocyte population is uniform with respect to proliferative potential and the ability to undergo neoplastic transformation. Further understanding of the hepatocyte population may have major scientific and medical impacts on treatment and management of patients with chronic HBV infection and may further our understanding of the pathways to neoplastic transformation and the development of HCC.
